# Altered Bioenergetics of Blood Cell Sub-Populations in Acute Pancreatitis Patients

**DOI:** 10.3390/jcm8122201

**Published:** 2019-12-13

**Authors:** Jack C. Morton, Jane A. Armstrong, Ajay Sud, Alexei V. Tepikin, Robert Sutton, David N. Criddle

**Affiliations:** 1 Department of Cellular and Molecular Physiology, Institute of Translational Medicine, University of Liverpool, Liverpool L69 3BX, UK; jack.morton87@gmail.com (J.C.M.); kiev@liverpool.ac.uk (A.V.T.); 2 Department of Clinical Cancer Medicine, Institute of Translational Medicine, University of Liverpool, Liverpool L69 3BX, UK; janearm@liverpool.ac.uk (J.A.A.); suda@liverpool.ac.uk (A.S.); sutton@liv.ac.uk (R.S.)

**Keywords:** acute pancreatitis, mitochondrial dysfunction, Seahorse bioenergetics, respiration, glycolysis, inflammation, leukocytes

## Abstract

Acute pancreatitis (AP) is a debilitating, sometimes fatal disease, marked by local injury and systemic inflammation. Mitochondrial dysfunction is a central feature of pancreatic damage in AP, however, its involvement in circulating blood cell subtypes is unknown. This study compared mitochondrial bioenergetics in circulating leukocytes from AP patients and healthy volunteers: 15 patients with mild to severe AP were compared to 10 healthy controls. Monocytes, lymphocytes and neutrophils were isolated using magnetic activated cell sorting and mitochondrial bioenergetics profiles of the cell populations determined using a Seahorse XF24 flux analyser. Rates of oxygen consumption (OCR) and extracellular acidification (ECAR) under conditions of electron transport chain (ETC) inhibition (“stress” test) informed respiratory and glycolytic parameters, respectively. Phorbol ester stimulation was used to trigger the oxidative burst. Basal OCR in all blood cell subtypes was similar in AP patients and controls. However, maximal respiration and spare respiratory capacity of AP patient lymphocytes were decreased, indicating impairment of functional capacity. A diminished oxidative burst occurred in neutrophils from AP patients, compared to controls, whereas this was enhanced in both monocytes and lymphocytes. The data demonstrate important early alterations of bioenergetics in blood cell sub-populations from AP patients, which imply functional alterations linked to clinical disease progression.

## 1. Introduction

Acute pancreatitis (AP) is a multifaceted disease, caused predominantly by gallstones and alcohol excess, which involves local injury and systemic inflammation. In severe disease, this may develop into a systemic inflammatory response syndrome, remote organ injury and death of the patient. The incidence of AP is 13–45 per 100,000 cases per year, and imposes a significant healthcare burden [1,2]. However, there is an incomplete understanding of the underlying pathophysiology, with current predictors of disease outcome inadequate and no specific therapy available. Damage to the pancreatic acinar cell is considered the initiating event of AP, manifested by premature zymogen activation, vacuolisation, mitochondrial dysfunction and necrotic cell death [3,4]. Bile acids, non-oxidative ethanol metabolites, and cholecystokinin hyperstimulation disrupt acinar cell calcium signalling and induce mitochondrial damage, via opening of the mitochondrial permeability transition pore (MPTP), causing loss of membrane potential, rundown of nicotinamide adenine dinucleotide (NADH), and fall of adenosine triphosphate (ATP) production, leading to necrosis [5,6,7,8,9,10].

However, comparatively little is known about mitochondrial dysfunction in circulating blood cells during AP. This may partly reflect the significant challenge posed by the isolation of patient blood cells in a reliable state for such bioenergetics measurements. In recent years, there has been increasing focus on the roles of immune cell subsets in the systemic inflammatory response in AP [11,12]. For example, neutrophil infiltration is evident in the pancreas within minutes of the onset of AP and exerts a significant role in disease severity [11,13]. Previously, elevated mitochondrial respiration was reported in a total population of peripheral blood mononuclear cells obtained from patients with mild AP, suggesting inefficient mitochondria, although no alteration of ATP production occurred [14]. However, whether specific bioenergetics alterations occur in blood cell subtypes during clinical AP is currently unknown. Detailed investigations of mitochondrial dysfunction can be achieved by measuring bioenergetics changes in cell populations using Seahorse flux analysis [15,16]. We have recently demonstrated that oxidative stress, which is elevated in clinical AP [17], altered the bioenergetic profiles of isolated pancreatic acinar cells determining cell death patterns [18]. 

In the present study, we have investigated the bioenergetics profiles of leukocyte sub-types isolated from AP patient blood samples, comparing results to those obtained from healthy volunteers. Our data show distinct alterations of mitochondrial bioenergetics in blood cell sub-types that occur during early clinical AP, pointing to a modified functional capacity of circulating blood cells during the inflammatory response.

## 2. Experimental Section

### 2.1. Blood Collection and Cell Isolation

Patients aged ≥18 years with a first attack of acute pancreatitis were recruited on the day of admission to the Royal Liverpool University Hospital for donation of blood and linked clinical data into the National Institute for Health Research Liverpool Pancreas Biomedical Research Unit Acute Pancreatitis Biobank, as approved by the regional ethics committee (REC 10/H1308/31 and 15/YH/0193). Patients with acute pancreatitis of any aetiology with two of three diagnostic features (serum amylase ≥3× upper limit of normal, typical pain, pancreatic inflammation on cross-sectional imaging) with written informed consent were eligible for inclusion, but patients who were unable to consent, had a history of recurrent acute or chronic pancreatitis or a history of pancreatic surgery or malignancy were excluded. Samples were collected prospectively within 24 h of admission from consenting patients who had presented within 72 h of onset of pain, together with clinical data that allowed severity stratification according to the 2012 Revised Atlanta Classification [19] after discharge. Blood samples were also collected from healthy volunteers (control group) aged ≥18 years; individuals with diabetes or a history of pancreatic disease were excluded. Collection, processing, storage, monitoring and usage of samples followed pre-defined standard operating procedures adhering to Good Clinical Practice. 

Blood samples (one 8.5 mL/tube) were collected in a K2EDTA tube (Vacuette, Greiner Bio-One GmbH, Kremsmünster, Austria) and processed within an hour of collection using an established protocol [19]. All isolation procedures were designed and carefully executed to prevent activation of blood cells. In brief, following collection blood samples were centrifuged at 500× *g* (acceleration 6 and no brake; Thermo Fisher Scientific, Waltham, MA, USA), the buffy layer removed and diluted with RPMI-140 (Sigma, Poole, UK) to 24 mL, then applied to a Histopaque density gradient (specific gravity 1.077/1.113, at room temperature; Alere, Waltham, MA, USA) and centrifuged at 700× *g* (acceleration 6, no brake and at room temperature; Thermo Fisher Scientific, Waltham, MA, USA). Three distinct bands were present; the uppermost band contained peripheral blood mononuclear cells (PBMCs), the middle band polymorphonuclear cells (PMNs) and the lower band contained red blood cells (RBCs). The PBMCs and PMNs were collected separately. Red cell lysis buffer (Sigma, Poole, UK) was added to the PMNs, improving the purity of the cell population by lysing the RBCs. 

The mononuclear cells were suspended in 80 µL of MACs buffer (PBS, 2 mM EDTA and 0.5% BSA; pH 7.2 and sterile filtered) and 20 µL CD61 human microbeads (Miltenyi, Bergisch Gladbach, Germany) at 4 ˚C for 15 min. The CD61 microbeads, which bind to CD61+ platelets, were then applied to a MS column (Miltenyi, Bergisch Gladbach, Germany) in a MiniMACS magnet (Miltenyi, Bergisch Gladbach, Germany) according to manufacturer’s instructions. The column was discarded (removing any platelets from the PBMCs) and the flow through collected and re-suspended in 80 µL of MACs buffer and 20 µL CD14 human microbeads (Miltenyi, Bergisch Gladbach, Germany). CD14+ monocytes were purified from the PBMC fraction using superparamagnetic iron-dextran microbead-labelled anti-CD14 antibodies. Cells retained in the column were collected by elution with MACs buffer after removal from the magnetic field. Lymphocytes, in comparison, were present in the through flow. Isolation yielded cell populations with >90% purity and viability as determined by fluorescence-activated cell sorting and Trypan Blue exclusion, respectively (Table S1).

### 2.2. Assessment of Monocyte, Lymphocyte and Neutrophil Bioenergetics

Purified monocytes, lymphocytes and neutrophils were re-suspended in XF assay buffer (Dulbecco’s Modified Eagle Medium (DMEM), 2 mM sodium pyruvate, 2 mM L-Glutamine and 10 mM D-glucose in ddH2O, pH 7.4 and sterile filtered), and then plated (250,000 cells/well) in 200 μL on CellTak (BD Biosciences, Poole, UK) coated assay plates and allowed to attach for 30 min at 37 ˚C in a non-CO_2_ incubator. The cellular bioenergetics of the isolated cells were determined using the XF24 analyser (Agilent, Boston, MA, USA) [18,19,20]. Real-time, non-invasive measurements of OCR and ECAR were obtained which correlated to mitochondrial function and glycolysis, respectively. Using the mitochondrial respiratory function “stress” test protocol, inhibitors of the mitochondrial electron transport chain (ETC) (oligomycin, 0.5 µg/mL; carbonyl cyanide-4-trifluoromethoxy phenylhydrazone (FCCP), 0.6 µM; rotenone and antimycin, 1 µM; Sigma, Poole, UK) and an activator of the oxidative burst (phorbol 12-myristate 13-acetate (PMA), 100 ng/mL; Sigma, Poole, UK) were sequentially injected to assess the following respiratory parameters: oxygen consumption rate (OCR) basal respiration, maximal respiration, spare respiratory capacity ATP turnover capacity, proton leak, non-mitochondrial respiration, and PMA-induced oxidative burst, extracellular acidification rate (ECAR) baseline, glycolytic reserve and PMA-induced ECAR. 

The mean basal respiration was determined at the 5th OCR measurement, before addition of the inhibitors or activators. ATP turnover capacity and proton leak were determined following injection of oligomycin, which blocks the ATP synthase, and then maximal respiration following FCCP, an uncoupler of the electron transport chain. The difference between the basal OCR and maximal OCR represents the Spare Respiratory Capacity OCR of the mitochondria. Antimycin A, an inhibitor of Complex III, and rotenone, an inhibitor of Complex I, were used in conjunction to completely inhibit mitochondrial electron transport: the remaining OCR is attributed to non-mitochondrial OCR. Basal OCR, proton leak OCR, and the maximal OCR were calculated after correction for the non-mitochondrial OCR for each assay. Finally, the oxidative burst OCR was determined cell following cell stimulation with PMA, a protein kinase C (PKC) activator that increases nicotinamide adenine dinucleotide phosphate (NADPH) oxidase activity. The ECAR measures were recorded in parallel to OCR measurements. Baseline ECAR was determined at the 5th ECAR reading and Glycolytic Reserve calculated by subtraction of the baseline ECAR reading from that obtained after addition of oligomycin. The optimal concentrations of the inhibitors and activator used for the assessment of mitochondrial function were as previously determined [21]. All XF assays were performed in sterile DMEM (5 mM D-glucose, 4 mM L-glutamine and 1 mM sodium pyruvate; pH 7.4). 

### 2.3. Statistics 

For each blood sample, 3–5 replicates were used for all bioenergetics determinations, and the data are presented as mean ± standard error of the mean (SEM). Statistical significance was determined using a Student’s t-test or Mann Whitney U test, with *p* ≤ 0.05 taken as indicating significant difference from control.

## 3. Results

### 3.1. Characteristics of Patients and Healthy Controls Included in the Analysis

For the study blood samples were collected from 15 AP patients. Of these, 12 were classified as mild AP, 1 was moderate and 2 patients had severe AP according to the revised Atlanta Classification [22]. The mean age of the patients was 57.2, with 11 females (mean age of 54.3 years) and 4 males (mean age of 65.3 years). The aetiology of AP in patients was: 12 biliary, 2 idiopathic, 1 alcoholic and 1 ERCP. Amylase, platelets and WBC counts (neutrophils, lymphocytes, monocytes, eosinophils and basophils) were recorded for each patient at admission by the hospital staff (Table 1). Further details of AP patient co-morbidities and Body Mass Index (BMI) are included in Table S2. For comparison, blood samples were collected from 10 healthy volunteers, of which half were male and half female. The overall mean age was 32.9 years, with female mean age of 34.4 years and male mean age of 31.4 years.

### 3.2. Bioenergetics Differences in OCR Between Healthy Volunteers and AP Patients

**Figure 1:**
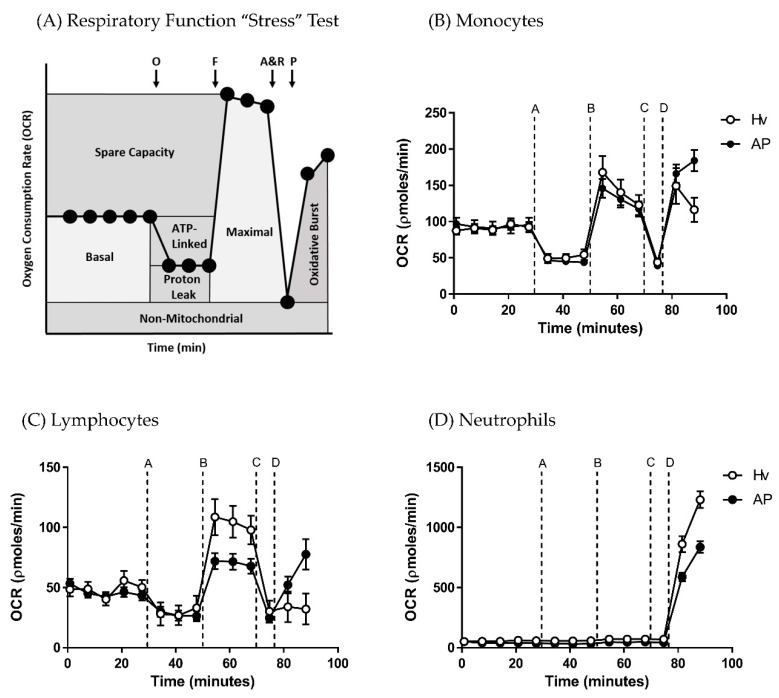
Bioenergetics changes in blood cell subtypes from acute pancreatitis (AP) patients and healthy volunteers (Hv). (**A**) Changes in oxygen consumption rates (OCR) with time in response to a mitochondrial respiratory function “stress” test using sequential applications of A = oligomycin 0.5 µg/mL, B = carbonyl cyanide-4-trifluoromethoxy phenylhydrazone (FCCP: F) 0.6 µM, C = antimycin (A) 1 µM and rotenone (R) 1 µM, and D = phorbol 12-myristate 13-acetate (P) 100 ng/mL to measure standard respiratory parameters in (**B**) monocytes, (**C**) lymphocytes and (**D**) neutrophils. Values are expressed as means ± standard error of the mean (SEM) with biological repeats of *N* = 10 (Hv) and 15 (AP).

Application of a mitochondrial respiratory function “stress” test protocol allowed measurement of standard respiratory parameters (basal respiration, maximal respiration, spare respiratory capacity, ATP turnover capacity, proton leak and non-mitochondrial respiration) in monocytes, lymphocytes and neutrophils, providing a comparison between AP patient and healthy volunteers. The protocol illustrated in Figure 1A shows OCR changes caused by sequential injection of mitochondrial inhibitors (oligomycin, FCCP, rotenone/antimycin), followed by an activator of the oxidative burst PMA used to derive comparative bioenergetics parameters. 

**Figure 2:**
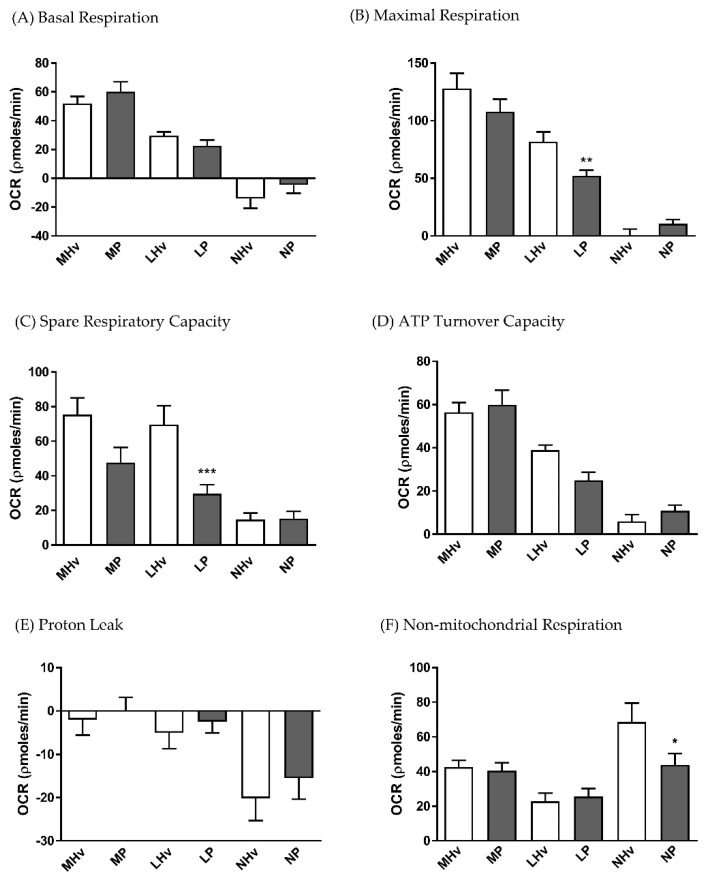
Mean changes in bioenergetics parameters determined in blood cell subtypes from acute pancreatitis (P) patients and healthy volunteers (Hv). Changes in oxygen consumption rates (OCR) obtained in monocytes (M), lymphocytes (L) and neutrophils (L) are shown in response to a mitochondrial respiratory function “stress” test to measure standard respiratory parameters (**A**–**F**). Values are expressed as means ± SEM with biological repeats of *N* = 10 (Hv) and 15 (AP). Significant changes in blood cells from AP patients compared to healthy controls are denoted as * *p* ≤ 0.05, ** *p* ≤ 0.01 and *** *p* ≤ 0.001. (MHv, LHv, NHv = monocytes, lymphocytes and neutrophils from healthy volunteers, respectively; MP, LP, NP = monocytes, lymphocytes and neutrophils from patients).

The blood cell sub-types exhibited distinct bioenergetics profiles (Figure 1B–D). The basal OCR values after 5 min equilibration were 51.92 ± 4.9 and 60.19 ± 6.9 in monocytes, 29.62 ± 2.6 and 22.54 ± 2.1 in lymphocytes, and −14.19 ± 6.7 and −4.6 ± 5.7 pmol/min in neutrophils in healthy volunteers and AP patients, respectively. There were no significant differences in basal respiration between AP patient and healthy volunteers for any of the blood cell types. However, when the “stress” test was applied, differences in bioenergetics were revealed. Thus, changes of OCR induced by inhibition of the electron transport chain showed that lymphocytes from AP patients exhibited a substantially decreased maximal respiration (Figure 2B; *p* ≤ 0.001) and spare respiratory capacity (Figure 2C; *p* ≤ 0.001) compared to lymphocytes from healthy controls. 

There was a trend for reduced spare respiratory capacity in monocytes from AP patients compared to healthy volunteers, although this did not attain significance (Figure 2C). Furthermore, no significant differences in ATP turnover capacity (Figure 2D) or proton leak (Figure 2E) were detected between AP patient and healthy control blood cells. However, a significantly reduced non-mitochondrial respiratory component was found in AP patient neutrophils compared to controls (Figure 2F).

### 3.3. Analysis of Mitochondrial Bioenergetics Differences in ECAR Between Healthy Volunteers and AP Patients

**Figure 3:**
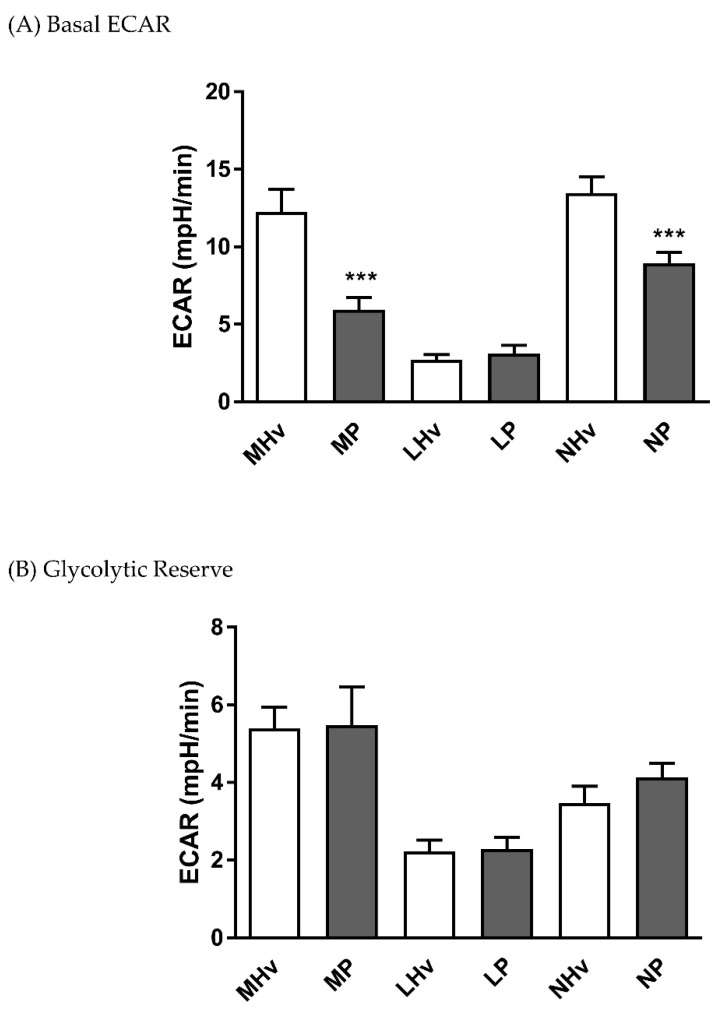
Mean changes in bioenergetics parameters determined in blood cell subtypes from acute pancreatitis patients and healthy volunteers. Changes in extracellular acidification rates (ECAR) obtained in monocytes (M), lymphocytes (L) and neutrophils (L) from patients (P) and healthy volunteers (Hv) are shown on basal glycolysis (**A**) and in response to mitochondrial inhibition to measure glycolytic reserve (**B**). Values are expressed as means ± SEM with biological repeats of *N* = 10 (Hv) and 15 (AP). Significant changes in blood cells from AP patients compared to healthy controls are denoted as *** *p* ≤ 0.001.

The basal ECAR values after 5 min equilibration were 12.27 ± 1.4 and 5.94 ± 0.8 in monocytes, 2.69 ± 0.4 and 3.11 ± 0.5 in lymphocytes, and 13.47 ± 1.1 and 8.92 ± 0.7 mpH/min in neutrophils in healthy volunteers and AP patients, respectively. Both monocyte and neutrophil basal ECARs were significantly decreased in AP patients compared to their respective healthy volunteer controls (Figure 3A; *p* ≤ 0.001). However, no significant differences in glycolytic reserve, measured as the change in ECAR following application of oligomycin, were apparent in any blood cell sub-type. 

### 3.4. Analysis of the Oxidative Burst in Healthy Volunteers and AP Patients 

**Figure 4:**
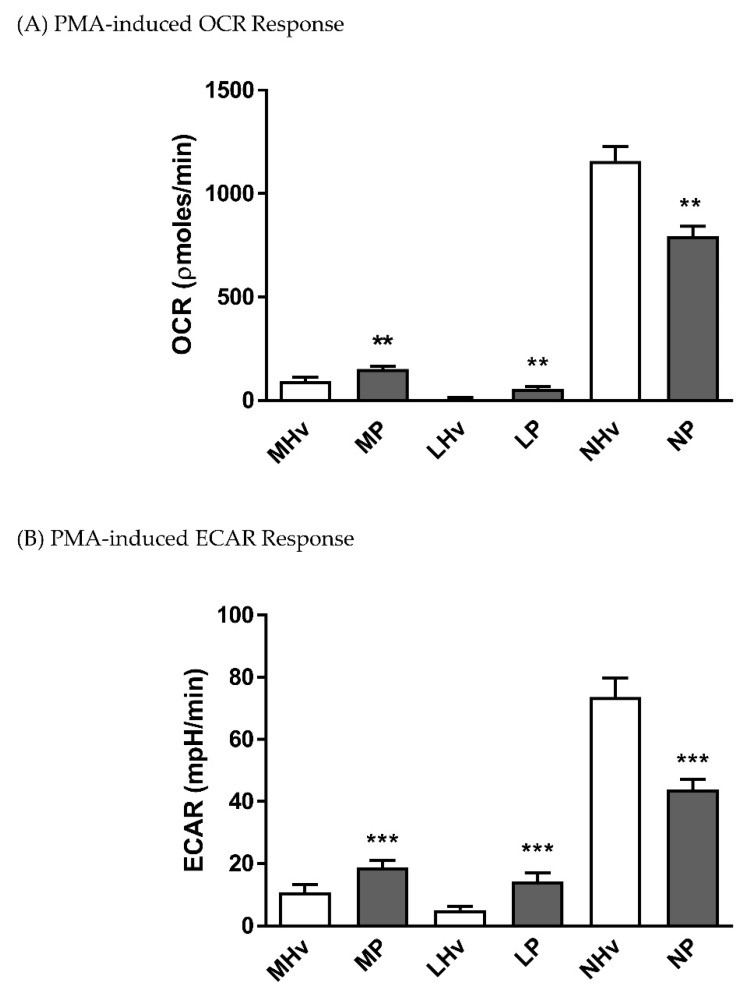
Mean changes in bioenergetics linked to the oxidative burst in blood cell subtypes from acute pancreatitis (P) patients and healthy volunteers (Hv). Changes in (**A**) oxygen consumption rates (OCR) and (**B**) extracellular acidification rates (ECAR) obtained in monocytes (M), lymphocytes (L) and neutrophils (L) in response to phorbol 12-myristate 13-acetate (PMA) to measure bioenergetics changes during the oxidative burst (**A**,**B**). Values are expressed as means ± SEM with biological repeats of *N* = 10 (Hv) and 15 (AP). Significant changes in blood cells from AP patients compared to healthy controls are denoted as ** *p* ≤ 0.01 and *** *p* ≤ 0.001.

Both monocytes and lymphocytes from AP patients exhibited significantly increased PMA-induced oxidative respiratory bursts (Figure 4A; *p* ≤ 0.01) and accompanying ECAR increases (Figure 4B; *p* ≤ 0.001) compared to those from healthy volunteers. In contrast, neutrophils from AP patients had a significantly decreased PMA-induced oxidative respiratory burst compared to healthy volunteer neutrophils (Figure 4A; *p* ≤ 0.001), mirrored by a reduced PMA-induced ECAR increase (Figure 4B; *p* ≤ 0.001).

## 4. Discussion

This study has demonstrated distinct alterations of mitochondrial bioenergetics in blood cell sub-types that occur during clinical AP, pointing to a modified functional capacity during the inflammatory response. Differences between patient and healthy volunteer blood cell respiration were only apparent, however, following manipulation of the ETC or stimulation with PMA, with no significant differences in basal OCR detected. Relatively little is known about bioenergetics changes in circulating blood cells that occur during clinical AP. Previously, a study showed that leukocytes from mild AP patients exhibited an approximate 1.5 fold elevation of endogenous respiration compared to controls with no associated change in ATP production [14]. This reflected a summation of activity in a population of peripheral blood mononuclear cells rather than changes in discrete subsets. Our study now indicates that bioenergetics profiles of monocytes, lymphocytes and neutrophils undergo complex alterations during the early course of AP, with subset-specific changes that do not simply reflect a generalized depression of mitochondrial activity. Thus, lymphocyte respiration was diminished in AP patients, with substantially reduced maximal respiration and spare respiratory capacity, whereas these parameters were unaltered in monocytes and neutrophils. 

Previously, it has been suggested that prospective monitoring of lymphocyte signalling profiles might assist predicting AP outcome: a variety of alterations in severe alcoholic AP patients with organ dysfunction was detected, linked to increased infection risk and sustained inflammation [23]. Our findings in lymphocytes from predominantly mild AP patients show a markedly reduced spare respiratory capacity. This bioenergetic parameter is considered an important indication of mitochondrial capacity to meet metabolic demands under stress conditions and is decreased in pathophysiological situations, including cardiac and neurodegenerative damage [24,25,26]. Common features of disease progression may be present in inflammatory states, which are reflected by alterations of blood cell subtype bioenergetics profiles. For example, similarities between inflammation in AP and sepsis have been reported [27], with defects in oxidative metabolism found in leukocytes from sepsis patients, including a substantial reduction of maximum oxygen consumption [28]. A compromise of lymphocyte function in AP linked to altered bioenergetic capacity would likely increase as the disease progresses and cellular stress augments. Oxidative stress is elevated in AP patients, coupled with a decrease of antioxidant capacity [17]. Oxidative stress strongly modified pancreatic acinar cell bioenergetics, thereby determining local cell fate [18,20], while mitochondrial bioenergetic function of human peripheral blood leucocytes was susceptible to oxidative injury, an effect that was greater in aged individuals [29]. Interestingly, mitochondrial dynamics have recently been shown to modulate lymphocyte fate through metabolic programming linked to bioenergetics changes. Thus, in activated effector T cells, which mediate protective immunity against pathogens, fission-dependent cristae expansion was associated with reduced ETC efficiency and promotion of aerobic glycolysis [30]. Although our study found a decreased maximal respiration in AP patient lymphocytes, this was not associated with a fall in ATP turnover capacity, suggesting maintenance of basal function. Accordingly, there was no associated increase in basal glycolysis, implying a defect of oxidative phosphorylation that did not trigger a switch in metabolic pathway. In agreement, no significant alteration of ATP production was detected in peripheral blood mononuclear cells from mild AP patients [14], while peripheral blood mononuclear cells from septic paediatric patients, which had a significantly reduced spare respiratory capacity, exhibited no differences in basal or ATP-linked oxygen consumption [31].

Our study demonstrated a diminished oxidative burst in neutrophils from AP patients, with significantly reduced OCR and ECAR responses to PMA stimulation compared to controls. Neutrophils play an important role in the early phase of AP, participating in digestive enzyme activation and progression to severe disease [13]. They utilise an oxidative burst via the activation of NADPH oxidase, which consumes oxygen and forms superoxide radicals [32]. Accordingly, depletion of neutrophils was protective in mild and severe experimental AP induced by caerulein [33] and by taurocholate [34], respectively. Alterations of neutrophil bioenergetics have been reported in other diseases. For example, changes in neutrophil oxidative bursts have been associated with autoimmune diseases such as multiple sclerosis, arthritis and recurrent infection [19], while diminution of neutrophil activity involving a reduced oxidative burst in response to formyl peptides, impaired phagocytosis and associated ROS production may underlie an increased susceptibility to bacterial infection in elderly individuals [35]. In AP patient neutrophils, both baseline ECAR and non-mitochondrial OCR were also reduced: the sum of the bioenergetics alterations would indicate that normal activity of neutrophils is compromised in AP patients, or alternatively, that at the time of measurement these immune cells had already performed their inflammatory role. A future study including measurement of neutrophil bioenergetics changes at multiple time-points would assist clarification. 

In contrast, an increased oxidative burst was detected in both monocytes and lymphocytes from AP patients compared to controls. This supports monocyte/macrophage involvement as a principal, important feature of early events in AP [11]. For example, blockade of monocyte chemoattractant protein synthesis was protective against experimental AP in mice [36], while application of antibodies against macrophage migration inhibitory factor improved AP survival in rats [37]. The increased oxidative burst capacity detected in AP patients may reflect an upregulation of NADPH oxidases in circulating monocytes, a feature which occurs in acute respiratory distress syndrome in response to ethanol [38]. Marked elevations of mitochondrial superoxide have recently been reported in peripheral blood mononuclear cells from mild AP patients [14]. Monocytes and macrophages are part of the innate immune system and exhibit a high degree of plasticity. Activated pro-inflammatory macrophages (M1) release copious amounts of cytokines, including interleukin 6 (IL6), IL12, IL1β and tumour necrosis factor alpha (TNFα), early in the inflammatory response and in AP make a significant contribution to the systemic inflammatory response syndrome linked to organ dysfunction and death [12]. Recently extra-acinar protease activation within macrophages during endocytosis of zymogen-containing vesicles has been shown to participate in the systemic inflammatory response and determine AP severity [39]. In our study, the PMA-induced ECAR response of AP patient monocytes was also greater than those of healthy volunteers, indicating an enhanced glycolytic component of ATP production during the respiratory burst with prioritisation of cellular oxygen to generate free radicals. A concurrent decrease of basal ECAR in monocytes was also found, implying a reduced glycolytic component contributing to basal energy production during AP, although the basis for this is presently unclear. 

Previously separation of blood cell subtypes for evaluation of mitochondrial function has been questioned since this may increase time before performing the assays [14] and potentially disrupt cellular interactions necessary for cell activation [40]. Here, we have shown that successful isolation and separation of AP patient blood cells is achievable for detailed bioenergetics investigations: basal OCR values were not different between AP patient and control groups for all subtypes, indicating no detrimental changes due to cell isolation procedures. Alterations of mitochondrial function have been observed with aging [41] and a limitation of the present study is that the healthy volunteer group was younger than the AP group. However, the subset-specific changes detected did not appear to simply reflect a generalized depression of mitochondrial activity that might be expected as a consequence of aging, and point to more precise changes: our results demonstrated differential alterations of bioenergetics linked to the oxidative burst in leukocyte subtypes from AP patients, and further revealed an important reduction of respiratory capacity in AP patient lymphocytes. These changes occurred early in the development of clinical AP, advancing our understanding of pathophysiological events in the inflammatory response. Detection of specific bioenergetics alterations of blood cell subtypes from patient samples may provide a more detailed picture of on-going mitochondrial dysfunction during AP and potentially assist prediction of outcome. 

## 5. Conclusions

Our data show distinct alterations of mitochondrial bioenergetics in blood cell sub-types that occur during early clinical AP, pointing to a modified functional capacity of circulating blood cells during the inflammatory response.

## Figures and Tables

**Table 1:** Demographics, aetiology and blood cell details of acute pancreatitis (AP) patients obtained on admission for the study.

Patient	Severity (1 = Mild, 2 = Moderate & 3 = Severe)	Aetiology (ERCP = Endoscopic Retrograde Cholangiopancreatography)	Sex (M = Male, F = Female)	Age	Amylase	Platelets (×10 > 9/L, *N*: 150–400)	White Blood Cells (×10 > 9/L, N: 3.5–11)	Neutrophils (×10 > 9/L, *N*: 2.0–7.5)	Lymphocytes (×10 > 9/L, *N*: 1.0–3.5)	Monocytes (×10 > 9/L, *N*: 0.2–0.8)	Eosinophils (×10 > 9/L, *N*: 0.0–0.4)	Basophils (×10 >9/L, *N*: 0.0–0.2)
**AP779**	1	Biliary	M	59	2634	228	10.8	8.3	1.5	0.8	0.1	0.1
**AP784**	3	Idiopathic	F	77	1240	293	25.6	22.7	1.4	1.5	0	0.1
**AP785**	1	Biliary	M	74	527	233	19.4	17.3	0.6	1.3	0.1	0
**AP788**	1	Biliary	F	20	1692	197	12.3	8.7	2.2	1.3	0	0
**AP796**	1	Biliary	F	64	1485	246	13.1	10.2	1.6	1.2	0	0
**AP797**	1	Biliary	M	63	2168	245	11.3	10.2	0.4	0.6	0	0
**AP799**	1	Biliary	F	28	1968	278	8.9	7	1.4	0.5	0	0.1
**AP805**	2	Biliary	F	80	1577	303	10.3	9.1	0.8	0.3	0	0
**AP806**	1	Alcohol	M	65	1265	188	10.7	8	1.3	1.2	0.1	0.1
**AP812**	1	Idiopathic	F	21	492	194	12	6.7	4.1	1	0.2	0
**AP821**	1	Biliary	F	33	2333	335	12.9	9.9	1.9	1	0.1	0.1
**AP828**	1	Biliary	F	77	1792	237	23	21.4	1.3	0.2	0.1	0.1
**AP837**	3	Biliary	F	91	1450	190	12.4	11.8	0.4	0.3	0	0
**AP839**	1	Biliary	F	49	2293	264	9.5	6	2.5	0.9	0.1	0
**AP842**	1	ERCP	F	57	2184	229	10.5	9.6	0.6	0.3	0	0

## Supplementary Materials

The following are available online at https://www.mdpi.com/2077-0383/8/12/2201/s1, Table S1: The isolation of blood cell populations obtained >90% (A) purity and (B) viability, as determined by fluorescence-activated cell sorting and Trypan Blue exclusion, respectively; Table S2: Details of (A) co-morbidities and (B) BMI from AP patients.

## References

[1] Peery A.F., Crockett S.D., Murphy C.C., Lund J.L., Dellon E.S., Williams J.L., Jensen E.T., Shaheen N.J., Barritt A.S., Lieber S.R. (2019). Burden and cost of gastrointestinal, liver, and pancreatic diseases in the united states: Update 2018. Gastroenterology.

[2] Yadav D., Lowenfels A.B. (2013). The epidemiology of pancreatitis and pancreatic cancer. Gastroenterology.

[3] Kruger B., Albrecht E., Lerch M.M. (2000). The role of intracellular calcium signaling in premature protease activation and the onset of pancreatitis. Am. J. Pathol..

[4] Raraty M., Ward J., Erdemli G., Vaillant C., Neoptolemos J.P., Sutton R., Petersen O.H. (2000). Calcium-dependent enzyme activation and vacuole formation in the apical granular region of pancreatic acinar cells. Proc. Natl. Acad. Sci. USA.

[5] Voronina S.G., Barrow S.L., Simpson A.W., Gerasimenko O.V., da Silva Xavier G., Rutter G.A., Petersen O.H., Tepikin A.V. (2010). Dynamic changes in cytosolic and mitochondrial atp levels in pancreatic acinar cells. Gastroenterology.

[6] Criddle D.N., Murphy J., Fistetto G., Barrow S., Tepikin A.V., Neoptolemos J.P., Sutton R., Petersen O.H. (2006). Fatty acid ethyl esters cause pancreatic calcium toxicity via inositol trisphosphate receptors and loss of atp synthesis. Gastroenterology.

[7] Booth D.M., Murphy J.A., Mukherjee R., Awais M., Neoptolemos J.P., Gerasimenko O.V., Tepikin A.V., Petersen O.H., Sutton R., Criddle D.N. (2011). Reactive oxygen species induced by bile acid induce apoptosis and protect against necrosis in pancreatic acinar cells. Gastroenterology.

[8] Mukherjee R., Mareninova O.A., Odinokova I.V., Huang W., Murphy J., Chvanov M., Javed M.A., Wen L., Booth D.M., Cane M.C. (2016). Mechanism of mitochondrial permeability transition pore induction and damage in the pancreas: Inhibition prevents acute pancreatitis by protecting production of atp. Gut.

[9] Wen L., Voronina S., Javed M.A., Awais M., Szatmary P., Latawiec D., Chvanov M., Collier D., Huang W., Barrett J. (2015). Inhibitors of orai1 prevent cytosolic calcium-associated injury of human pancreatic acinar cells and acute pancreatitis in 3 mouse models. Gastroenterology.

[10] Criddle D.N. (2016). Reactive oxygen species, ca(2+) stores and acute pancreatitis; a step closer to therapy?. Cell Calcium.

[11] Xue J., Sharma V., Habtezion A. (2014). Immune cells and immune-based therapy in pancreatitis. Immunol. Res..

[12] Mayerle J., Sendler M., Hegyi E., Beyer G., Lerch M.M., Sahin-Toth M. (2019). Genetics, cell biology, and pathophysiology of pancreatitis. Gastroenterology.

[13] Gukovskaya A.S., Vaquero E., Zaninovic V., Gorelick F.S., Lusis A.J., Brennan M.L., Holland S., Pandol S.J. (2002). Neutrophils and nadph oxidase mediate intrapancreatic trypsin activation in murine experimental acute pancreatitis. Gastroenterology.

[14] Chakraborty M., Hickey A.J., Petrov M.S., Macdonald J.R., Thompson N., Newby L., Sim D., Windsor J.A., Phillips A.R. (2016). Mitochondrial dysfunction in peripheral blood mononuclear cells in early experimental and clinical acute pancreatitis. Pancreatology.

[15] Brand M.D., Nicholls D.G. (2011). Assessing mitochondrial dysfunction in cells. Biochem. J..

[16] Kramer P.A., Chacko B.K., George D.J., Zhi D., Wei C.C., Dell’Italia L.J., Melby S.J., George J.F., Darley-Usmar V.M. (2015). Decreased bioenergetic health index in monocytes isolated from the pericardial fluid and blood of post-operative cardiac surgery patients. Biosci. Rep..

[17] Tsai K., Wang S.S., Chen T.S., Kong C.W., Chang F.Y., Lee S.D., Lu F.J. (1998). Oxidative stress: An important phenomenon with pathogenetic significance in the progression of acute pancreatitis. Gut.

[18] Armstrong J.A., Cash N.J., Ouyang Y., Morton J.C., Chvanov M., Latawiec D., Awais M., Tepikin A.V., Sutton R., Criddle D.N. (2018). Oxidative stress alters mitochondrial bioenergetics and modifies pancreatic cell death independently of cyclophilin d, resulting in an apoptosis-to-necrosis shift. J. Biol. Chem..

[19] Kramer P.A., Chacko B.K., Ravi S., Johnson M.S., Mitchell T., Darley-Usmar V.M. (2014). Bioenergetics and the oxidative burst: Protocols for the isolation and evaluation of human leukocytes and platelets. J. Vis. Exp..

[20] Armstrong J.A., Cash N.J., Morton J.C., Tepikin A.V., Sutton R., Criddle D.N. (2019). Mitochondrial targeting of antioxidants alters pancreatic acinar cell bioenergetics and determines cell fate. Int. J. Mol. Sci..

[21] Chacko B.K., Kramer P.A., Ravi S., Johnson M.S., Hardy R.W., Ballinger S.W., Darley-Usmar V.M. (2013). Methods for defining distinct bioenergetic profiles in platelets, lymphocytes, monocytes, and neutrophils, and the oxidative burst from human blood. Lab. Investig..

[22] Banks P.A., Bollen T.L., Dervenis C., Gooszen H.G., Johnson C.D., Sarr M.G., Tsiotos G.G., Vege S.S., Acute Pancreatitis Classification Working Group (2013). Classification of acute pancreatitis—2012: Revision of the atlanta classification and definitions by international consensus. Gut.

[23] Oiva J., Mustonen H., Kylanpaa M.L., Kyhala L., Kuuliala K., Siitonen S., Kemppainen E., Puolakkainen P., Repo H. (2010). Acute pancreatitis with organ dysfunction associates with abnormal blood lymphocyte signaling: Controlled laboratory study. Crit. Care.

[24] Sansbury B.E., Jones S.P., Riggs D.W., Darley-Usmar V.M., Hill B.G. (2011). Bioenergetic function in cardiovascular cells: The importance of the reserve capacity and its biological regulation. Chem. Biol. Interact..

[25] Yadava N., Nicholls D.G. (2007). Spare respiratory capacity rather than oxidative stress regulates glutamate excitotoxicity after partial respiratory inhibition of mitochondrial complex i with rotenone. J. Neurosci..

[26] Kramer P.A., Darley-Usmar V.M. (2015). The emerging theme of redox bioenergetics in health and disease. Biomed. J..

[27] Kylanpaa M.L., Repo H., Puolakkainen P.A. (2010). Inflammation and immunosuppression in severe acute pancreatitis. World J. Gastroenterol..

[28] Cheng S.C., Scicluna B.P., Arts R.J., Gresnigt M.S., Lachmandas E., Giamarellos-Bourboulis E.J., Kox M., Manjeri G.R., Wagenaars J.A., Cremer O.L. (2016). Broad defects in the energy metabolism of leukocytes underlie immunoparalysis in sepsis. Nat. Immunol..

[29] Tsai K., Hsu T.G., Lu F.J., Hsu C.F., Liu T.Y., Kong C.W. (2001). Age-related changes in the mitochondrial depolarization induced by oxidative injury in human peripheral blood leukocytes. Free Radic. Res..

[30] Buck M.D., O’Sullivan D., Klein Geltink R.I., Curtis J.D., Chang C.H., Sanin D.E., Qiu J., Kretz O., Braas D., van der Windt G.J. (2016). Mitochondrial dynamics controls t cell fate through metabolic programming. Cell.

[31] Weiss S.L., Selak M.A., Tuluc F., Perales Villarroel J., Nadkarni V.M., Deutschman C.S., Becker L.B. (2015). Mitochondrial dysfunction in peripheral blood mononuclear cells in pediatric septic shock. Pediatr. Crit. Care Med..

[32] Karlsson A., Nixon J.B., McPhail L.C. (2000). Phorbol myristate acetate induces neutrophil nadph-oxidase activity by two separate signal transduction pathways: Dependent or independent of phosphatidylinositol 3-kinase. J. Leukoc. Biol..

[33] Sandoval D., Gukovskaya A., Reavey P., Gukovsky S., Sisk A., Braquet P., Pandol S.J., Poucell-Hatton S. (1996). The role of neutrophils and platelet-activating factor in mediating experimental pancreatitis. Gastroenterology.

[34] Abdulla A., Awla D., Thorlacius H., Regner S. (2011). Role of neutrophils in the activation of trypsinogen in severe acute pancreatitis. J. Leukoc. Biol..

[35] Sauce D., Dong Y., Campillo-Gimenez L., Casulli S., Bayard C., Autran B., Boddaert J., Appay V., Elbim C. (2017). Reduced oxidative burst by primed neutrophils in the elderly individuals is associated with increased levels of the cd16bright/cd62ldim immunosuppressive subset. J. Gerontol. A Biol. Sci. Med. Sci..

[36] Bhatia M., Ramnath R.D., Chevali L., Guglielmotti A. (2005). Treatment with bindarit, a blocker of mcp-1 synthesis, protects mice against acute pancreatitis. Am. J. Physiol. Gastrointest. Liver Physiol..

[37] Sakai Y., Masamune A., Satoh A., Nishihira J., Yamagiwa T., Shimosegawa T. (2003). Macrophage migration inhibitory factor is a critical mediator of severe acute pancreatitis. Gastroenterology.

[38] Yeligar S.M., Harris F.L., Hart C.M., Brown L.A. (2012). Ethanol induces oxidative stress in alveolar macrophages via upregulation of nadph oxidases. J. Immunol..

[39] Sendler M., Weiss F.U., Golchert J., Homuth G., van den Brandt C., Mahajan U.M., Partecke L.I., Doring P., Gukovsky I., Gukovskaya A.S. (2018). Cathepsin b-mediated activation of trypsinogen in endocytosing macrophages increases severity of pancreatitis in mice. Gastroenterology.

[40] Schmid D., Burmester G.R., Tripmacher R., Kuhnke A., Buttgereit F. (2000). Bioenergetics of human peripheral blood mononuclear cell metabolism in quiescent, activated, and glucocorticoid-treated states. Biosci. Rep..

[41] Sun N., Youle R.J., Finkel T. (2016). The mitochondrial basis of aging. Mol. Cell.

