# Supplementary material for: Altered Bioenergetics of Blood Cell Sub-Populations in Acute Pancreatitis Patients
Source: J Clin Med. 2019 Dec 13;8(12):2201. doi: 10.3390/jcm8122201 (PMC6947319; doi:10.3390/jcm8122201)
Supplement: Supplementary file 1 [file jcm-08-02201-s001.pdf]

**Table S1.** The isolation of blood cell populations obtained >90% (**A**) purity and (**B**) viability, as determined by fluorescence-activated cell sorting and Trypan Blue exclusion, respectively.

(A)

| | Monocyte (CD14+) | Lymphocyte (CD14-) | Neutrophils |
|---|---|---|---|
| Monocyte (%) | 94.27 | 0.35 | 1.4 |
| Lymphocyte (%) | 0.70 | 97.30 | 97.2 |
| Neutrophil (%) | 1.10 | 2.40 | 0.2 |

(B)

| Individual | Monocyte Total | Monocyte Alive | Monocyte Alive (%) | Lymphocyte Total | Lymphocyte Alive | Lymphocyte Alive (%) | Neutrophil Total | Neutrophil Alive | Neutrophil Alive (%) |
|---|---|---|---|---|---|---|---|---|---|
| Patient 1 | 2150000 | 1967500 | 91.5 | 6500000 | 6110000 | 94 | 31500000 | 30555000 | 97.0 |
| Patient 2 | 1800000 | 1683000 | 93.5 | 10450000 | 9663500 | 92.5 | 21000000 | 19950000 | 95.0 |
| Patient 3 | 3300000 | 3004000 | 91.0 | 8400000 | 7686000 | 91.5 | 28000000 | 26040000 | 93.0 |
| Patient 4 | 1900000 | 1786000 | 94.0 | 14000000 | 13440000 | 96.0 | 18000000 | 17460000 | 97.0 |
| Patient 5 | 2150000 | 1956000 | 91.0 | 7200000 | 6624000 | 92.0 | 21000000 | 19010000 | 90.5 |
| Patient 6 | 1500000 | 1447500 | 96.5 | 6900000 | 6486000 | 94.0 | 27000000 | 26460000 | 98.0 |
| Patient 7 | 1800000 | 1656000 | 92.0 | 9050000 | 8597500 | 95.0 | 24000000 | 23040000 | 96.0 |
| Patient 8 | 2550000 | 2397000 | 94.0 | 9400000 | 8648000 | 92.0 | 25000000 | 23375000 | 93.5 |
| Patient 9 | 2400000 | 2280000 | 95.0 | 13000000 | 12545000 | 96.5 | 30500000 | 27450000 | 90.0 |
| Patient 10 | 3300000 | 3168000 | 96.0 | 10500000 | 10185000 | 97.0 | 33000000 | 30690000 | 93.0 |
| Patient 11 | 2850000 | 2793000 | 98.0 | 9800000 | 9212000 | 94.0 | 29000000 | 28130000 | 97.0 |
| Patient 12 | 2500000 | 2437000 | 97.5 | 8600000 | 7912000 | 92.0 | 29000000 | 26970000 | 93.0 |
| Patient 13 | 345000 | 327750 | 95.0 | 8100000 | 7614000 | 94.0 | 26000000 | 25090000 | 96.5 |
| Patient 14 | 3100000 | 2867500 | 92.5 | 13000000 | 11830000 | 91.0 | 28000000 | 26880000 | 96.0 |
| Patient 15 | 2750000 | 2626000 | 95.5 | 12000000 | 11760000 | 98.0 | 26000000 | 23920000 | 92.0 |

| | | | | | | | | | |
|---|---|---|---|---|---|---|---|---|---|
| Control 1 | 1550000 | 1457000 | 94.0 | 6350000 | 6159500 | 97.0 | 16000000 | 15435000 | 96.5 |
| Control 2 | 2100000 | 2016000 | 96.0 | 4800000 | 4560000 | 95.0 | 17000000 | 16240000 | 95.5 |
| Control 3 | 1400000 | 1351000 | 96.5 | 7100000 | 6958000 | 98.0 | 23000000 | 22310000 | 97.0 |
| Control 4 | 1700000 | 1564000 | 92.0 | 8300000 | 8175500 | 98.5 | 14500000 | 13630000 | 94.0 |
| Control 5 | 1850000 | 1739000 | 94.0 | 5850000 | 5674500 | 97.0 | 19500000 | 19305000 | 99.0 |
| Control 6 | 2100000 | 2016000 | 96.0 | 6200000 | 5952000 | 96.0 | 24000000 | 23520000 | 98.0 |
| Control 7 | 2100000 | 2005500 | 95.5 | 9200000 | 8832000 | 96.0 | 21000000 | 20370000 | 97.0 |
| Control 8 | 1800000 | 1773000 | 98.5 | 3700000 | 3478000 | 94.0 | 21000000 | 20265000 | 96.5 |
| Control 9 | 1550000 | 1519000 | 98.0 | 6100000 | 5734000 | 94.0 | 18000000 | 16920000 | 94.0 |
| Control 10 | 2405000 | 2212650 | 92.0 | 5550000 | 5383500 | 97.0 | 16000000 | 14960000 | 93.5 |

**Table S2.** Details of (**A**) co-morbidities and (**B**) BMI from AP patients.

(A)

| Patient | Charlson Comorbidity Index (CCI) | Co-morbidities |
|---|---|---|
| AP779 | 3 | Type 2 Diabetes, asthma |
| AP784 | 9 | Type 2 Diabetes Mellitus, hypertension, Chronic Kidney Disease, Chronic Obstructive Airways Disease, Hiatus Hernia, Osteoarthritis |
| AP785 | 5 | Benign Prostatic Hyperplasia, Chronic Obstructive Airways Disease (Home Nebuliser), Hypertension, Giant Cell Arteritis |
| AP788 | 0 | |
| AP796 | 2 | Cervical Cancer (in remission after Total Abdominal Hysterectomy) |
| AP797 | 2 | Benign Prostatic Hyperplasia |
| AP799 | 0 | |
| AP805 | 4 | Hypertension, Hypothyroidism |
| AP806 | 5 | Alcoholic Liver Disease (Liver Cirrhosis) |
| AP812 | 0 | |
| AP821 | 0 | Depression |
| AP828 | 7 | Hypertension, Type 2 Diabetes Mellitus, Chronic Kidney Disease, Osteoarthritis |
| AP837 | 5 | Hypertension, Peptic Ulcer Disease, Osteoarthritis |
| AP839 | 1 | Anxiety and Depression |
| AP842 | 2 | |

(B)

| **Patient** | **BMI (Kg/$m^2$)** |
|---|---|
| AP779 | 38.94 |

| | |
|---|---|
| AP784 | 27.22 |
| AP785 | 28.72 |
| AP788 | 31.93 |
| AP796 | 21 |
| AP797 | 24.99 |
| AP799 | 22.90 |
| AP805 | 36.52 |
| AP806 | 29.48 |
| AP812 | Not available |
| AP821 | Not available |
| AP828 | 33.00 |
| AP837 | 23.39 |
| AP839 | 41.52 |
| AP842 | 21.55 |
